# Trait‐based climate vulnerability of native rodents in southwestern Mexico

**DOI:** 10.1002/ece3.6323

**Published:** 2020-05-18

**Authors:** Arturo Ramírez‐Bautista, James H. Thorne, Mark W. Schwartz, John N. Williams

**Affiliations:** ^1^ Centro Interdisciplinario de Investigación para el Desarrollo Integral Regional Unidad Oaxaca (CIIDIR‐OAX) Instituto Politécnico Nacional Oaxaca Mexico; ^2^ Department of Environmental Science & Policy University of California Davis CA USA

**Keywords:** climate model, endemism, Oaxaca, rare species, small mammal, spatial analysis, threat, vegetation type

## Abstract

**Aim:**

Incorporate species’ trait information together with climate projections for associated habitat to assess the potential vulnerability of rodent taxa to climate change.

**Location:**

Oaxaca State, Mexico.

**Methods:**

We used a trait‐based approach together with climate exposure models to evaluate the vulnerability of rodent species to projected climate conditions in the study region. Vulnerability was estimated based on three factors: (a) Level of climatic exposure that species are projected to experience across their current statewide range; (b) inherent species‐specific sensitivity to stochastic events; and (c) species’ capacity to cope with climate change effects. We defined species as inherently sensitive if they had any of the following: restricted geographic distribution in Mexico; narrow altitudinal range; low dispersal ability; or long generation length.

**Results:**

Vulnerability varied depending on the climate change scenario applied. Under the MPI general circulation model and current emissions trends, by 2099, all species evaluated were projected to have some level of threat (vulnerable for at least one factor), with 4 out of 55 species vulnerable for all three factors, 29 for two factors, and 22 for one factor. Six out of ten rodent species endemic to Oaxaca were vulnerable for two or more factors. We found that species with narrow and restricted‐range distributions combined with low adaptive capacity were projected to be particularly vulnerable.

**Main conclusions:**

By including species‐specific trait information in climate exposure assessments, researchers can contextualize and enhance their understanding about how climate change is likely to affect individual taxa in an area of interest. As such, studies like this one provide more relevant threat assessment information than exposure analyses alone and serve as a starting point for considering how climatic changes interact with an array of other variables to affect native species across their range.

## Graphical abstract

We used a trait‐based approach together with climate exposure models to evaluate the potential vulnerability of rodent species to climate change in Oaxaca, the most mammal‐diverse state in Mexico. Depending on the climate scenario, most to all species were projected to have some level of vulnerability with narrow and restricted range species projected to be at the highest risk.

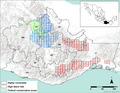

## 1 INTRODUCTION

Human‐induced climate change is considered one of the major threats to the world´s biodiversity (Parmesan & Yohe, 2003; Thomas et al., 2004), with widespread predictions of species’ range shifts and extinctions (Javeline et al., 2015; MacLean & Beissinger, 2017). Many effects of climate change on biodiversity have already been seen worldwide (Parmesan, 2006), and evaluating these effects has become a crucial task for researchers and conservationists (Aguirre et al., 2017; Khaliq, Hof, Prinzinger, Böhning‐Gaese, & Pfenninger, 2014; Markovic, Carrizo, Kärcher, Walz, & David, 2017; Mohammadi, Ebrahimi, Moghadam, & Bosso, 2019; Ribeiro, Sales, De Marco, & Loyola, 2016; Widick & Bean, 2019).

Researchers have employed a range of ecological niche modelling techniques (Choe, Thorne, & Seo, 2016; Elith et al., 2006; Phillips & Dudík, 2008; Santos & Cheylan, 2013; Thorne et al., 2013; Williams et al., 2009) as one way to evaluate the impact of climate change on species distributions. In this approach, species´ geographic ranges are modelled into the future based on the projected extent and distribution of the climatic conditions (and/or other niche variables) associated with their current or historic ranges (Willis et al., 2015). Although range modelling is useful for identifying where species may need to move to track suitable conditions, the uncertainty of climate predictions and the lack of incorporation of species´ biological data have driven researchers to look for more integrated approaches (Stewart et al., 2015; Urban et al., 2016; Williams, Shoo, Isaac, Hoffmann, & Langham, 2008).

Trait‐based vulnerability assessments (TVAs) rely on the assumption that species are likely to respond to climate change in different ways depending on their specific biological attributes (Böhm et al., 2016; Williams et al., 2008). TVAs often consider some measure of climate exposure, frequently expressed as the degree of change of environmental conditions under different climate change scenarios. In this study, we conducted a TVA in the context of a climate exposure analysis, considering a combination of characteristics (e.g., biological, ecological, and behavioral) that could make a species more or less sensitive, or bestow it with greater or lesser adaptive capacity to climate change effects (Foden et al., 2013; Willis et al., 2015).

A major challenge for TVAs is finding adequate data to quantify specific attributes for individual species. One way to deal with this challenge is to use more widely available proxy variables that provide roughly the same information as the more direct, but more difficult to obtain, metric in question. In the case of this study, for example, we used body mass as a proxy for climatic (i.e., thermal) tolerance. Similarly, to estimate inherent adaptability, we used generation time as a proxy for evolutionary rate of change, where shorter generation lengths confer more opportunities for heritable adaptations per unit time (Foden et al., 2013). Additionally, we used species distribution range as a proxy for genetic variation, where larger ranges imply greater landscape variability and as a result may correlate with greater genotypic/phenotypic diversity (Urban et al., 2016).

TVAs have been employed at global and continental scales to produce vulnerability assessments for groups of organisms, such as mammals and amphibians (Dickinson, Orme, Suttle, & Mace, 2014). In addition to identifying organisms of concern, this approach identifies regions for conservation priority, albeit at coarse scales. In this study, we combined those two applications of TVA by assessing climate change vulnerability of the mammalian order Rodentia at a regional scale in the Nearctic–Neotropical transition zone of southwestern Mexico—one of the more speciose and topographically diverse regions of Mesoamerica (Garcia‐Mendoza, Ordoñez, & Briones‐Salas, 2004; Myers, Mittermeier, Mittermeier, da Fonseca, & Kent, 2000; Ortega & Arita, 1998).

Specifically, our study looks at species vulnerability along three axes: (a) climate sensitivity based on ecological attributes; (b) adaptive capacity based on biological attributes; and (c) climatic change across a species’ geographic range. While a species’ conservation status may be negatively affected by any one of these factors, species that are vulnerable according to two or even all three factors are presumably at elevated risk. Thus, just as Rabinowitz (1981) presented a framework in which species could be triply rare based on range, habitat specificity, and local abundance, we consider the potential vulnerability of Oaxaca's rodent species based on climate sensitivity, adaptive capacity, and level of exposure to climate change—where species that are vulnerable for multiple factors face greater risk of decline or extinction.

## 2 MATERIALS AND METHODS

### 2.1 Study area

**FIGURE 1:**
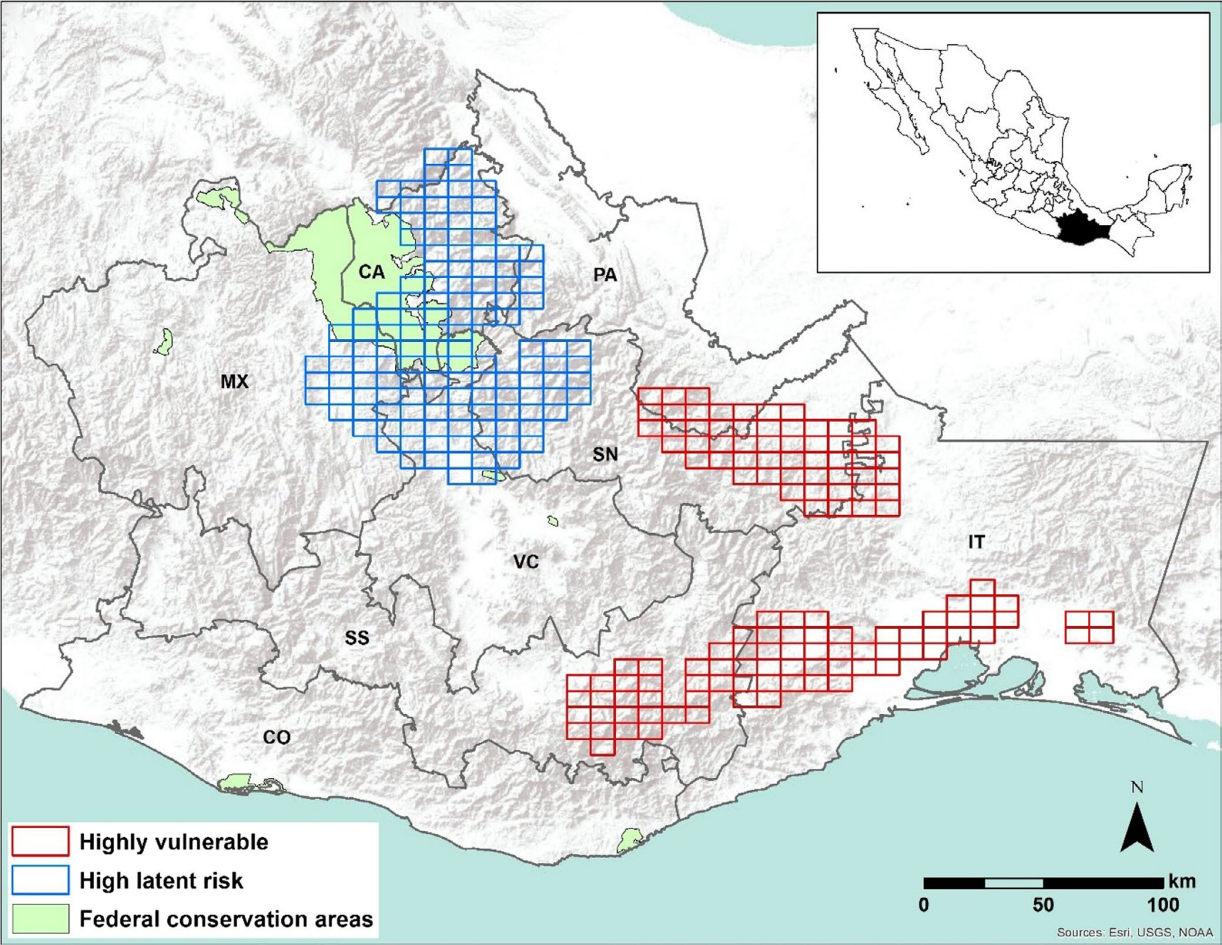
Map of Oaxaca State (inset shows location in Mexico) identifies where rodent species are projected to be at different levels of future climate‐related risk. Red cells represent sites where at least two highly vulnerable species are present; blue cells represent sites where at least two high latent risk species are present (see Methods for definitions). The major geographic–socioeconomic regions are depicted by gray lines: Isthmus of Tehuantepec (IT); Costa de Oaxaca (CO); Valles Centrales (CV); Sierra Sur (SS); Sierra Norte (SN); Cañada (CA); Mixteca (MX); and Papaloapan (PA). Light green polygons represent federally protected natural areas

The state of Oaxaca is located in southwestern Mexico (Figure 1) and represents roughly 5% (93,757 km^2^) of the national territory. With 43.5% of Mexico´s mammal species (Briones‐Salas, Cortés‐Marcial, & Lavariega, 2015), it is a major contributor to the biodiversity of the country and of the Mesoamerica hotspot (Myers et al., 2000) due to high degrees of plant and animal endemism, and despite significant habitat loss and degradation. After bats, rodents are the second most diverse mammal taxon, accounting for 30% of the State´s mammal diversity (Briones‐Salas et al., 2015).

### 2.2 Rodent data

There are 64 native rodent species recorded in Oaxaca (Briones‐Salas et al., 2015). We excluded species with unconfirmed distributions or insufficient data according to Ceballos and Oliva (2005) and Ceballos, Blanco, González, and Martínez (2006). This left 55 species (10 endemic to Oaxaca) belonging to 24 genera and seven families (Table 1). The families represented occupy a broad range of ecological niches and include: pocket gophers (Geomyidae) and kangaroo rats/mice (Heteromyidae); agoutis (Dasyproctidae), pacas (Cuniculidae), and New World porcupines (Erethizontidae) from the caviomorpha infraorder; squirrels (Sciuridae); and New World mice, rats, and voles (Cricetidae). Species’ altitudinal range data were obtained from the Mexican Commission for the Knowledge and Use of Biodiversity (CONABIO; www.conabio.gob.mx/informacion/gis/). The maps are based on species distribution models made using the Genetic Algorithm for Rule‐set Prediction (GARP), and on maps from the Atlas of Mexican Mammals (Ceballos et al., 2006).

**TABLE 1:** Rodent species native or endemic (*) to the state of Oaxaca, Mexico with accompanying recorded altitudinal ranges according to Ceballos and Oliva (2005)

Species	Family	Min altitude	Max altitude	Altitude range
*Baiomys musculus*	Cricetidae	0	2,000	2,000
*Coendou mexicanus*	Erethizontidae	0	2,350	2,350
*Cuniculus paca*	Cuniculidae	0	1,800	1,800
*Dasyprocta mexicana*	Dasyproctidae	50	650	600
*Dipodomys phillipsii*	Heteromyidae	900	2,850	1,950
*Glaucomys volans*	Sciuridae	840	3,040	2,200
Habromys chinanteco*	Cricetidae	2,080	2,650	570
Habromys ixtlani*	Cricetidae	2,500	3,000	500
Habromys lepturus*	Cricetidae	2,500	3,000	500
*Heteromys desmarestianus*	Heteromyidae	45	1,860	1,815
*Liomys irroratus*	Heteromyidae	0	3,050	3,050
*Liomys pictus*	Heteromyidae	0	2045	2,045
Megadontomys cryophilus*	Cricetidae	2,400	3,500	1,100
*Megadontomys thomasi*	Cricetidae	3,000	3,500	500
*Microtus mexicanus*	Cricetidae	2,220	4,115	1,895
Microtus oaxacensis*	Cricetidae	1,500	2,500	1,000
*Microtus quasiater*	Cricetidae	700	2,150	1,450
Microtus umbrosus*	Cricetidae	1,700	2,400	700
*Neotoma mexicana*	Cricetidae	0	4,045	4,045
*Nyctomys sumichrasti*	Cricetidae	0	1,500	1,500
*Oligoryzomys fulvescens*	Cricetidae	0	1,550	1,550
Orthogeomys cuniculus*	Geomyidae	0	30	30
*Orthogeomys grandis*	Geomyidae	0	1,700	1,700
*Orthogeomys hispidus*	Geomyidae	0	2,360	2,360
*Oryzomys alfaroi*	Cricetidae	860	2,350	1,490
*Oryzomys chapmani*	Cricetidae	1,550	2,500	950
*Oryzomys couesi*	Cricetidae	0	2,300	2,300
*Oryzomys melanotis*	Cricetidae	0	2000	2,000
*Oryzomys rostratus*	Cricetidae	0	1,500	1,500
*Peromyscus aztecus*	Cricetidae	500	3,200	2,700
*Peromyscus difficilis*	Cricetidae	1,200	3,700	2,500
*Peromyscus furvus*	Cricetidae	650	2,950	2,300
*Peromyscus gratus*	Cricetidae	1,710	2,700	990
*Peromyscus leucopus*	Cricetidae	0	3,000	3,000
*Peromyscus maniculatus*	Cricetidae	60	3,800	3,740
*Peromyscus megalops*	Cricetidae	1,500	3,000	1,500
Peromyscus melanocarpus*	Cricetidae	900	2,800	1,900
*Peromyscus melanophrys*	Cricetidae	50	2,700	2,650
Peromyscus melanurus*	Cricetidae	700	1900	1,200
*Peromyscus mexicanus*	Cricetidae	600	2000	1,400
*Reithrodontomys fulvescens*	Cricetidae	0	2,600	2,600
*Reithrodontomys megalotis*	Cricetidae	0	4,000	4,000
*Reithrodontomys mexicanus*	Cricetidae	90	1,800	1,710
*Reithrodontomys microdon*	Cricetidae	2,225	3,050	825
*Reithrodontomys sumichrasti*	Cricetidae	800	3,200	2,400
Rheomys mexicanus*	Cricetidae	0	2,200	2,200
*Sciurus aureogaster*	Sciuridae	0	3,300	3,300
*Sciurus deppei*	Sciuridae	0	2,800	2,800
*Scotinomys teguina*	Cricetidae	1,000	2,940	1,940
*Sigmodon alleni*	Cricetidae	0	3,050	3,050
*Sigmodon hispidus*	Cricetidae	0	3,050	3,050
*Sigmodon leucotis*	Cricetidae	1,800	2,623	823
*Sigmodon mascotensis*	Cricetidae	0	2,550	2,550
*Spermophilus variegatus*	Sciuridae	0	3,600	3,600
*Tylomys nudicaudus*	Cricetidae	0	1,600	1,600

### 2.3 Climate change vulnerability assessment

A study by Williams, Rivera, Choe, Schwartz, and Thorne (2018) modelling the effects of climate change on Oaxaca projected high levels of climate exposure for many of the vegetation types used by mammals. We used the climate exposure projections of Oaxaca's major vegetation types from that study as the basis for evaluating statewide habitat exposure for the respective rodent species evaluated. Overall species‐specific vulnerability to climate change was estimated based on three criteria: sensitivity to potential climate change; inherent adaptive capacity to climate variability (Glick, Stein, & Edelson, 2011); and climate exposure of associated habitat.

For each criterion, we selected a threshold that determined whether a species was at risk in that category or not. Such thresholds are by nature somewhat arbitrary, given most organisms show continuous, nonbinary response curves to most environmental cues. That said, the selection of appropriate thresholds is a critical part of developing a useful TVA approach that allows the method to be used by resource managers and conservation practitioners (Willis et al., 2015). In each of the sections below, we document the source and the justification for the respective threshold used so that readers may evaluate (and change as needed) the appropriateness of any given criterion for their own vulnerability assessment objectives.

#### 2.3.1 Sensitivity

In this study, sensitivity refers to the degree to which a species may be affected by anthropogenic or stochastic factors (Aguirre et al., 2017). We used three measures of sensitivity: species´ rarity; habitat suitability in Oaxaca; and altitudinal range. These criteria have been used previously for assessing the sensitivity of mammals and other groups to climate change (Böhm et al., 2016; Dickinson et al., 2014; Foden et al., 2013; Urban et al., 2016). The first two criteria are also considered in the Risk Evaluation Method (MER) developed by the Mexican Secretariat of Environment and Natural Resources (SEMARNAT) to designate priority conservation species nationwide.

Rare species are sensitive to stochastic events, as they typically have small populations, restricted distributions, or both (Arita, 1993; Foden et al., 2013). We used each species’ Mexico‐wide distribution as a proxy for rarity, where it was considered “highly sensitive” if its distribution comprised less than 5% of the national territory (SEMARNAT, 2010). While there are additional ways to define and evaluate rarity (e.g., Choe, Thorne, Hijmans, & Seo, 2019; Rabinowitz, 1981), the measure we used, combined with altitudinal breadth (see below), implicitly incorporates habitat specificity/breadth and overall, if not local, abundance inasmuch as these metrics may be correlated with broad‐scale geographic range (Brown, 1984).

We refer to habitat suitability as the percentage of a species´ distribution range that is considered favorable for in situ long‐term persistence (SEMARNAT, 2010). To obtain habitat suitability values, we overlaid each species´ distribution range on a land use and vegetation map (LaSorte & Jetz, 2010) using ArcMap v. 10.1 (www.esri.com) and calculated the percentage of the distribution inside unfavorable habitats. Unfavorable habitats were defined as human settlements, permanent agricultural fields (including cultivated grasslands), and bare lands. A species was considered “highly sensitive” if ≥60% of its distribution was located in unfavorable habitats (Estavillo, Pardini, & da Rocha, 2013; Huggett, 2005).

We used altitudinal range as a proxy for climate breadth, which is frequently considered among the variables that shape a species´ sensitivity to climate change (Böhm et al., 2016). Species with narrow elevational ranges are likely to be more sensitive than broadly distributed species due to the limited climatic breadth they are adapted to (Dickinson et al., 2014). A species was considered sensitive for this metric if it had an altitudinal range of ≤1,000 m (Santos & Cheylan, 2013).

#### 2.3.2 Adaptive capacity

Adaptive capacity refers to the ability of a species to resist, recover from, or adjust to stochastic events based on its biological attributes (Aguirre et al., 2017; Dickinson et al., 2014). For measures of adaptive capacity, we used species´ weight and generation length. In mammals, weight is generally positively related to dispersal ability and thermal tolerances—traits that are considered indicative of adaptability, given that high dispersal ability allows an organism to track suitable habitat over long distances, and thermal tolerance allows it to endure large temperature fluctuations (Schloss, Nuñez, & Lawler, 2012; Sutherland, Harestad, Price, & Lertzman, 2000). We considered a species to have low adaptive capacity if mean adult weight was ≤40 g (values obtained from Pacifici et al. (2013)).

Generation length (expressed in days) reflects the turnover rate of breeding individuals in a population (Pacifici et al., 2013) and integrates reproductive and demographic parameters such as age at first reproduction and lifespan. As such, generation length serves as a proxy for a species’ ability to react to stochastic events (Böhm et al., 2016) and as an indicator of evolutionary rate (Urban et al., 2016). Generation length values for each species were obtained from an online database (natureconservation.pensoft.net). We considered a species to have low adaptive capacity if it has a generation length ≥800 days; species with shorter generation lengths were considered to have average to better‐than‐average adaptive capacity.

#### 2.3.3 Climate exposure

As endothermic, small‐bodied mammals that are closely associated with habitat and microhabitat attributes, rodents are often considered to be minimally affected by the direct impacts (e.g., physiological stresses) of climate change (Buckley, Hurlbert, & Jetz, 2012; McNab, 2012). Instead, effects for this group are expected to be more associated with the response to climate change of the vegetation in their associated habitat (Cameron & Scheel, 2001; McCain & King, 2014; Santos, Thorne, & Moritz, 2015).

To account for this indirect relationship, we estimated climate exposure for the rodents of Oaxaca by looking at mid‐ and end‐century climate exposure projections for the vegetation associated with the range of each species. We used recently evaluated climate exposures for all vegetation types in Oaxaca at 1 × 1 km resolution (Williams et al., 2018). A baseline for present day climate exposure (based on precipitation and temperature) was calculated using a recent vegetation map (IEEDS, 2016; INEGI, 2010) and counting the frequency with which each vegetation type occupied a range of climate conditions for the period 1981–2010. The classification counts cells in the most frequently occupied 80% of climate conditions as “low” exposure or not stressed, from 80% to 95% frequency as uncertain or “medium” exposure, from 95% to 99% as “high” exposure, and the marginal 1% as “very high” exposure or stress (Thorne et al., 2017, 2018). Additionally, model projections yielded “nonanalog” cells—another type of very high exposure cell whose projected future temperature and precipitation values were without an analog in the baseline period. For each future climate scenario, we clipped the overall climate exposure map of Oaxaca to the range of each rodent species and summarized the proportion of climate exposure for the habitats it occupied.

We defined a rodent species’ climate exposure based on the proportion of its range in Oaxaca that was projected to be climatically stressed, including the exposure categories of “high”, “very high”, or “nonanalog.” We considered a species to be climatically exposed (i.e., climatically vulnerable) if ≥60% of the cells in its distribution in Oaxaca were projected to be in one of these high climatic stress categories. We used this threshold based on other studies that propose 30%–40% of historic range retention as the minimum amount of suitable habitat required for species persistence (Estavillo et al., 2013; Huggett, 2005).

The climate exposure values we used are based on Williams et al. (2018), who calculated them from five general circulation models (GCMs), including: CNRM – CM5 (Voldoire et al., 2013); GFDLCM3 (Donner et al., 2011); HADGEM2 – ES (Collins et al., 2011); MPI‐ESM – LR (Block & Mauritsen, 2013); and REA (Giorgi & Mearns, 2002). The models were run using two radiative forcing scenarios—RCP 4.5 (“low”) and RCP 8.5 (“current track”)—that are consistent with reduced and current CO_2_ emissions trends. The models were run for two future time periods: 2015–2039 (“near‐future”) and 2075–2099 (“end‐century”). These GCMs, concentration pathways and time periods were used here and by Williams et al. (2018) because at the time of writing they were what the Mexican government and the National Autonomous University's Center for Atmospheric Sciences proposed as the best projections and parameters for studies of climate change impact, vulnerability, and adaptation in Mexico (uniatmos.atmosfera.unam.mx/ACDM/). We present results for the “lower impact” (CNRM‐RCP 4.5) and “higher impact” (MPI‐RCP 8.5) scenarios, as these two combinations encompass the range of exposure projected by the other climate scenarios considered.

To analyze the magnitude of future exposure, we conducted a two‐tailed *t* test in R (R_Core_Team, 2017) to evaluate whether the mean percentage of a species’ range distribution projected to be under stressful conditions for a future climatic scenario represented a significant departure from baseline exposure levels.

#### 2.3.4 Vulnerability categories

We followed Foden et al. (2013) to classify each species into one of four climate change vulnerability categories: highly vulnerable (HV); potential adapters (PA); potential persisters (PP); and high latent risk (HLR)—depending on the combination of its climate sensitivity (in any of the following: rarity; habitat suitability; altitudinal range), adaptive capacity (average weight or generation length), and climate exposure. Potential adapters are those species with high exposure and sensitivity, but with average or better adaptive capacity. Potential persisters have high exposure and low adaptive capacity, but low sensitivity. Species in the high latent risk category are projected to have low exposure, but they present high sensitivity and low adaptive capacity, making them biologically susceptible species (Böhm et al., 2016). Species that did not enter into one these four categories were labelled as “exposed only”, “sensitive only”, “low adaptive capacity only”, or “low vulnerability” (not vulnerable according to any of the criteria; Table 2).

**TABLE 2:** Designation of climate change vulnerability categories used in this assessment. The first three columns refer to the vulnerability criteria that when marked by an ‘x’ indicate their inclusion as part of the associated vulnerability category (rows). Categories from Foden et al. (2013)

High exposure	High sensitivity	Low adaptive capacity	Vulnerability category
x	x	x	Highly Vulnerable (HV)
x		x	Potential Persisters (PP)
x	x		Potential Adapter (PA)
	x	x	High Latent Risk (HLR)
x			Exposed Only (EO)
	x		Sensitive Only (SO)
		x	Low Adaptive Capacity Only (LACO) Low Vulnerability (LV)
			Low Vulnerability (LV)

In analyzing the results, we also used a framework similar to that used for species rarity (Rabinowitz, 1981) to examine vulnerability along multiple axes.

## 3 RESULTS

A full breakdown by species of the vulnerability assessment according to the three vulnerability factors/axes is presented in appendices S1–S3 of the Supporting Information. Summaries and highlights of the assessment for each of the factors, and the species affected are presented below.

### 3.1 Climate sensitivity

Based on present day geographic range attributes and species characteristics, 21 of Oaxaca's rodent species (38%) were considered highly sensitive to potential changes in climate: 12 of them due to narrow altitudinal breadth (≤1,000 m); and 19 due to reduced distributional range in Mexico (≤ 5% of national territory). Currently, no species exhibit high sensitivity due to lack of suitable habitat because all had at least 40% of their distributional range in Oaxaca in favorable habitat (Appendix S1). All endemic species had high sensitivity with respect to distribution area at the national level, but only six were sensitive due to narrow altitudinal range (≤1,000 m): *Habromys chinanteco*; *H. ixtlani*; *H. lepturus*; *Microtus oaxacensis*; *M. umbrosus*; and *O. cuniculus*. In Oaxaca, the first five species are distributed on mountain tops and at mid‐elevations above 2,000 m a.s.l. in the Sierra Norte region. By contrast, *O. cuniculus* is only found in the lowlands of the Isthmus of Tehuantepec region at or below 30 m a.s.l.

### 3.2 Adaptive capacity

Overall, 38 rodent species (69%) showed low adaptive capacity to climate change effects: 16 due to long generation length (≥800 days); and 23 due to low body weight (≤40 g); *Peromyscus maniculatus* had low capacity in both categories. Five out of ten endemic species were included in this list: *Habromys chinanteco*; *H. ixtlani*; *Microtus oaxacensis*; *Orthogeomys cuniculus*; and *Rheomys mexicanus* (Appendix S1).

### 3.3 Climate exposure

**FIGURE 2:**
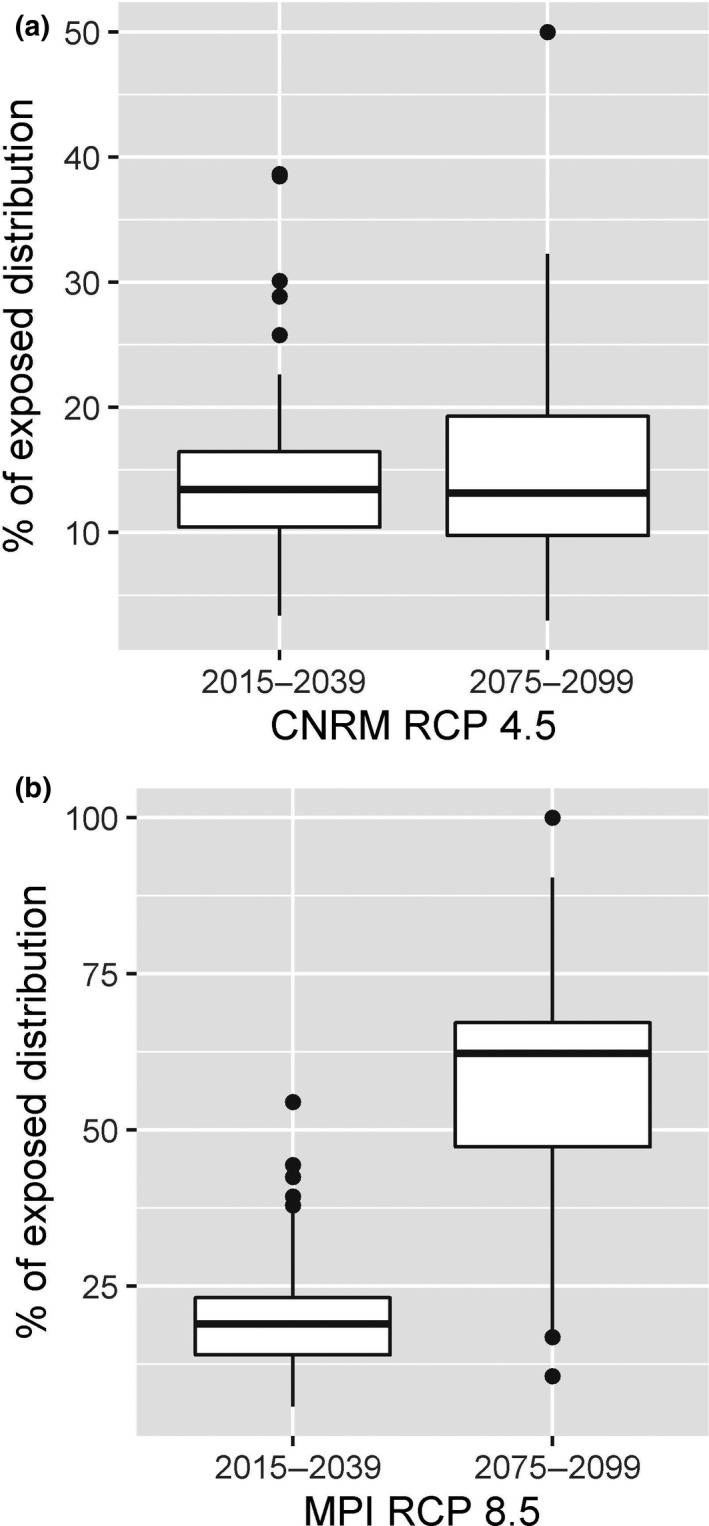
Percentage of species´ distribution range located in cells projected to be exposed (“high”, “very high”, and “nonanalog” categories in Williams et al.´s (2018) study) under the (a) lower impact (CNRM‐RCP 4.5) and (b) higher impact (MPI‐RCP 8.5) climate change scenarios, for two future periods: near‐future (2015–2039) and end‐century (2075–2099). Neither lower impact scenario represented a significant departure (*p* < .05) from baseline exposure, and only the end‐century projection for the higher impact scenario was significant (*p* < .001)

For the lower impact climate scenario (CNRM‐RCP 4.5), we found no significant difference between the mean percentage of grid cells of the species’ ranges projected to be under stressful conditions (“high”, “very high”, and “nonanalog” exposure categories) for the two future time periods compared to the baseline (*p* > .05) (Figure 2a, Appendix S2). By contrast, under the higher impact climate scenario (MPI‐RCP 8.5), there was a significant increase in the percentage of grid cells in the species’ ranges projected to be in the high exposure categories by end‐century compared to the baseline (*p* < .001) (Figure 2b). Endemic and nonendemic species were projected to be equally exposed for both climate change scenarios and future time periods.

No species was projected to be highly exposed (≥ 60% of grid cells in range projected to be in high exposure categories) under the CNRM‐RCP 4.5 scenario for either time period. By contrast, for the MPI‐RCP 8.5 scenario the number of highly exposed species went from zero for the near‐future period to 33 (60%) for the end‐century period, including two of the ten endemic species, *Orthogeomys cuniculus* and *Rheomys mexicanus*. *O. cuniculus* mainly inhabits seasonally dry tropical forests, while *R. mexicanus* is distributed in cloud forests, pine oak forests, and seasonally dry tropical forests.

### 3.4 Vulnerability

Overall species’ projected vulnerability as a function of the combined effects of climate sensitivity, adaptive capacity, and climate exposure varied considerably between the two impact scenarios. For the lower impact climate scenario, there was no difference between the number of species in different categories of vulnerability for the two future time periods (Table 3, Appendix S3), and no species fell into the categories of high vulnerability, potential persisters or potential adapters—the three highest risk categories. However, 13 species (23.6%) were classified as having high latent risk, that is, species that, while not projected to experience high climate exposure, are potentially sensitive to climate changes due to their biological attributes and may have minimal adaptive capacity under novel climate conditions.

**TABLE 3:** Number of rodent species projected to be in different categories of vulnerability using the lower impact (CNRM‐RCP 4.5) scenario and higher impact (MPI‐RCP 8.5) climate scenarios for near‐future (2015–2039) and end‐century (2075–2099) time periods in Oaxaca, Mexico. Percentage relative to all 55 species recorded in the state is presented in parentheses

Vulnerability	CNRM‐RCP 4.5		MPI‐RCP 8.5	
	Near‐future	End‐century	Near‐future	End‐century
Highly vulnerable	0	0	0	4 (7.3%)
Potential persisters	0	0	0	17 (31%)
Potential adapter	0	0	0	3 (5.5%)
High Latent Risk	13 (23.6%)	13 (23.6%)	13 (23.6%)	9 (16.4%)
Exposed only	0	0	0	10 (18.2%)
Sensitive only	8 (14.5%)	8 (14.5%)	8 (14.5%)	4 (7.3%)
Low adaptive capacity only	25 (45.5%)	25 (45.5%)	25 (45.5%)	8 (14.5%)
Low vulnerability	9 (16.4%)	9 (16.4%)	9 (16.4%)	0

**FIGURE 3:**
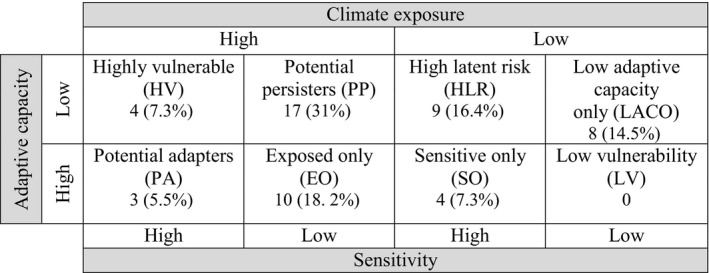
Number of rodent species projected to be in different categories of vulnerability using the higher impact (MPI‐RCP 8.5) climate scenario for end‐century (2075–2099) time period in Oaxaca, Mexico. Percentage relative to all 55 species recorded in the state is presented in parentheses

Figure 3 shows the breakdown by number of species into each of the vulnerability categories for the higher impact scenario. This scenario includes four species projected to be in the highest vulnerability category by the end‐century period: *Dasyprocta mexicana*; *Orthogeomys cuniculus*; *O. grandis*; and *Rheomys mexicanus*. Also, under this scenario, 17 species were classified as potential persisters (exposed with low adaptive capacity, but also with low sensitivity; Appendix S3). Three species fell into the potential adapters category (exposed species with high sensitivity, but with high adaptive capacity): *Sigmodon leucotis*; *Oryzomys chapmani*; and *Megadontomys thomasi*. Nine species fell into the category of high latent risk: *Habromys chinanteco*; *H. ixtlani*; *Microtus oaxacensis*; *M. quasiater*; *Peromyscus furvus*; *P. gratus*; *P. melanurus*; *Reithrodontomys microdon*; and *Scotinomys teguina*.

## 4 DISCUSSION

Studies using trait‐based assessments alongside climate projections provide greater context for evaluating species’ vulnerability than climate projections alone (Foden et al., 2013 for birds, amphibians, and corals; Dickinson et al., 2014 for amphibians and mammals; Böhm et al., 2016 for reptiles). This appears valid for rodent species in Oaxaca where, because they span a variety of physical and life history traits and occupy a range of niche strategies and habitat types, it is unlikely that climate alone will determine their long‐term viability (Pacifici et al., 2015). For example, we found that while 33 species (60%) were projected to be highly exposed (i.e., more than 60% of their distribution range was composed of highly exposed cells), only four (7%) scored as vulnerable in all three categories (sensitivity, adaptive capacity, and exposure) under the high‐impact scenario.

The task of identifying vulnerable species can thus be divided into the trait‐based assessment and the projected climate exposure components. The trait‐based assessment is subjective in that the results depend on which traits are chosen (see Methods for a justification of the traits we chose). Carefully selected, biologically relevant traits—as long as they are based on measurable parameters for which data exist—can nevertheless be robust and stable. By contrast, climate projections are more equivocal and dynamic—partly because they depend on multiple variables with complex interactions, and partly because conditions are constantly changing. We took this uncertainty into account by considering low‐ and high‐impact scenarios, understanding that these upper and lower bounds will likely change over time as models improve and conditions evolve.

With the above caveats in mind, we note that for the lower impact scenario (CNRM‐RCP 4.5) not only did no species meet the threshold for all three criteria, but relatively few (13) met two criteria, and none met the threshold for the climate exposure category for either the near‐future or end‐century projection periods. Thus, while most species were potentially vulnerable based on sensitivity, adaptive capacity, or both, no species was projected to experience climate‐related vulnerability under the parameters we chose. At face value this finding seems like a reason for optimism if society can reduce greenhouse gas emissions enough to put us on the RCP 4.5 track. Data from 2005 to 2015, however, suggest that we are currently headed toward the RCP 8.5 scenario (Hayhoe et al., 2017), so correcting our trajectory will require significant changes in emissions.

If we stay on the RCP 8.5 track and the higher impact MPI climate model proves accurate, four species (7.3%) are projected to be highly vulnerable by end‐century. While this number may seem small given more dire predictions (e.g., Urban et al., 2016), it is noteworthy that all 55 species are projected to be at risk for at least one criterion by end‐century, and relaxing threshold criteria slightly could push as many as 20 species (36%) into the high vulnerability category (Appendix S4).

With respect to the four highly vulnerable species, *Rheomys mexicanus* and *Orthogeomys cuniculus* are both restricted‐range Oaxaca endemics with international and Mexican federal protection status (SEMARNAT, 2010). Thus, these species are already at risk based on exogenous, nonclimate‐related factors. *R. mexicanus* is a small‐bodied, semi‐aquatic species of riparian forests and wetlands of the Sierra Sur and Isthmus of Tehuantepec that is listed as endangered by the IUCN (Timm, Álvarez‐Castañeda, & Lacher, 2018). *Orthogeomys cuniculus* is a medium‐sized species that is also limited to the lowlands of the Isthmus of Tehuantepec and is similarly identified as an IUCN species of concern because of decreasing population trends and deficient data (Castro‐Arellano & Vázquez, 2008). The large‐bodied (~5 kg) *D. mexicana* is also a restricted‐range species that, while not limited to Oaxaca, is nevertheless only found across parts of a few states in southern Mexico in tropical rain and cloud forests of the Sierra Norte and Isthmus of Tehuantepec (Figure 1)—habitats that are also at risk (Vázquez, Emmons, Reid, & Cuarón, 2008). The medium‐sized *O. grandis* is the only one of the four with a broader range—coastal forests and agricultural edges from the central Mexico state of Jalisco to southwestern Honduras—whose population is considered stable and of “least concern” according to IUCN (Vázquez, Emmons, & McCarthy, 2016). However, as neither Mexican nor IUCN conservation status listings explicitly incorporate climate risk, all four species merit re‐evaluation across their respective ranges.

### 4.1 Endemic and restricted‐range species vulnerability

In addition to *O. cuniculus* and *R. mexicanus*, four other endemic species were included in the category of high latent risk: *H. chinanteco*; *H. ixtlani*; *M. oaxacensis*; and *P. melanurus*. Four more were projected to be sensitive only: *Peromyscus melanocarpus*; *Microtus umbrosus*; *Megadontomys cryophilus*; and *Habromys lepturus*. Furthermore, all of the endemics except *O. cuniculus* and *R. mexicanus* are small‐sized rodents distributed at or near mountaintops above 1,500 m a.s.l., making their thermal adaptive capacity low, and greatly limiting their ability to move upslope in response to shifting climatic envelopes.

Range‐restricted species, especially those found on or near mountaintops, are considered to be among the most climate sensitive due to geographic isolation, limited mobility, and reductions in climatically suitable habitat (Ceballos, Rodriguez, & Medellin, 1998; LaSorte & Jetz, 2010; Parmesan, 2006; Schloss et al., 2012). Among the 55 species evaluated in this study, ten (18%) are distributed around mountaintops above 1,500 m a.s.l., but none of these fell into the high vulnerability category, and only five were projected to be at high latent risk: *H. chinanteco*; *H. ixtlani*; *M. oaxacensis*; *P. gratus*, and *R. microdon* (Appendix S4). By contrast, the two low‐elevation (below 1,000 m a.s.l.) restricted species (*D. mexicana* and *O. cuniculus*) were classified in the high vulnerability category.

Compared to mountaintop species, intermediate‐ and low‐elevation species are considered less vulnerable because they are often able to move uphill to encounter appropriate climatic conditions (Moritz et al., 2008; Parmesan, 2006). Such movements have been reported elsewhere for plants (Du et al., 2018; Lenoir, Gégout, Marquet, Ruffray, & Brisse, 2008; Leonelli, Pelfini, di Cella, & Garavaglia, 2011) and animals (Baltensperger & Huettmann, 2015; Hickling, Roy, Hill, Fox, & Thomas, 2006; Moritz et al., 2008; Tryjanowski, Sparks, & Profus, 2005).

For two of the four vulnerable species identified in this study, however, that movement is unlikely. *D. mexicana*, while able to disperse relatively long distances, shows a strong preference and specialization for humid tropical forests (below 800 m a.s.l.), where it can reach high densities (Ceballos & Oliva, 2005). Whether the vegetation it depends on would shift upslope with it or whether it could persist in cloud forests, which are typically adjacent upslope to tropical humid forests, is unclear. For *O. cuniculus*, although it is found exclusively at low elevations on the Isthmus of Tehuantepec (typically 0–30 m a.s.l.), its range may be limited as much by its fossorial behavior and need for specific soils (Emmons, 2016), as by any climatic factor related to a specific altitudinal range.

### 4.2 Conservation implications

Climate sensitivity, adaptive capacity, and climate exposure can be thought of as providing a three‐dimensional space in which to consider the climate‐related conservation priority of any species of concern. In this construct, each factor represents an axis or dimension that we classified as either low or high vulnerability based on selected thresholds, but that can also be considered along continua. Either way, continuum or threshold, we found that this multidimensional approach that considers both exogenous (climate, habitat availability) and endogenous (trait‐based) factors provides a more comprehensive way to evaluate overall climate vulnerability than a single‐factor method.

Rabinowitz (1981) argued convincingly that species that are rare in terms of geographic range, habitat specificity, and local abundance are at greater risk of extinction than species than species that rare in only one or two of those dimensions. Similarly, we propose that species with greater innate sensitivity to change, limited ability to adapt, and higher projected exposure are more climatically at risk than species with reduced vulnerability in one or more of those axes (Figure 3). We found that the most vulnerable species are distributed in the eastern part of the Sierra Norte and Sierra Sur regions and in the southwest part of the Isthmus of Tehuantepec (Figure 1). These areas consist mainly of tropical deciduous and evergreen forests, two of the most threatened ecosystems in Mexico and Oaxaca due to land use change (Corona, Galicia, Palacio‐Prieto, Bürgi, & Hersperger, 2016; Corona‐Núñez, Mendoza, & Galicia, 2018). Tropical ecosystems have long been recognized as a priority for conservation in Mexico and elsewhere due to their great diversity and endemism and because of high rates of loss or degradation (G. Ceballos et al., 1998; Lewis, Edwards, & Galbraith, 2015; Sánchez‐Cordero, Illoldi‐Rangel, Linaje, Sarkar, & Peterson, 2005). Recently, Williams et al. (2018) also found tropical ecosystems were projected to be the most climatically exposed vegetation types in Oaxaca.

Despite their conservation importance, tropical ecosystems are not well represented in the Natural Protected Areas (ANPs) of Oaxaca, which comprise only 5% of Oaxaca´s territory (Illoldi et al., 2008). Moreover, there is no range overlap between the existing ANPs and the four highly vulnerable species identified in this study, and only a small degree of overlap between ANPs and the range for the high latent risk species (Figure 1). With expansion of the federal protected areas network unlikely, there is a distinct need for conservation plans in areas such as those identified here to be designed and implemented with the participation of the local communities on whose land these ranges fall. Additionally, the scientific community together with state and federal governments could support these efforts with population monitoring of threatened species and climate change mitigation activities.

Species in the high latent risk category, also referred to as biologically susceptible (Böhm et al., 2016), are distributed in places that are not projected to have high climatic exposure. However, these species are classified as highly sensitive due to such factors as limited distributional range or suitable habitat, narrow climatic breadth, and low adaptive capacity. Most of these factors are related to biological attributes and thus relatively fixed through time (Foden et al., 2013), making their classification relatively constant compared to categories dependent on more variable criteria or the accuracy of the latest climate model (Nenzén & Araújo, 2011; Steen, Sofaer, Skagen, Ray, & Noon, 2017). That said, it is important to note that one of the factors determining latent risk—habitat suitability—is both sensitive to exogenous (i.e., human) forces and difficult to predict. Although habitat suitability was not a deciding variable in our evaluation because no species had ≥60% of its habitat compromised by human activity, this number is highly susceptible to change in the future (e.g., from deforestation or development), especially for limited range species for which small losses in absolute area may amount to large losses in percentage terms. Thus, of all the variables that go into our evaluation of overall species’ climate risk, habitat suitability is perhaps the one that most merits re‐evaluation on a regular basis.

The current suitability of habitat notwithstanding, we found the percentage of species with high latent risk to be relatively high (16%–24%) and affected little by the scenario or model used. As such (and for the habitat suitability concern mentioned above), we recommend that monitoring and conservation efforts include this group, as they are often characterized by traits such as restricted distribution/local endemism, low dispersal ability, and naturally vulnerable or sparsely occurring habitat such as mountaintops or cloud forests, respectively.

Finally, we recommend that government entities like the Secretariat of Natural Resources (SEMARNAT) consider formal integration of climate exposure and its interaction with species´ biological attributes into assessments of species´ extinction risk and vulnerability. Taking such action would force natural resource managers, scientists, and regulators to be explicit about the assumptions and criteria used to evaluate climate vulnerability and to design corresponding conservation strategies. The trait‐based vulnerability assessment approach presented in this study represents a promising and straightforward tool for achieving this objective in general, and for assessing species´ vulnerability in biologically diverse regions such as Oaxaca in particular.

## CONFLICT OF INTERESTS

The authors report no competing or conflicting interests with respect to publication of this manuscript.

## AUTHOR CONTRIBUTIONS

**Arturo Ramírez‐Bautista:** Formal analysis (lead); Writing‐original draft (lead). **James Thorne:** Conceptualization (equal); Methodology (equal); Writing‐review & editing (supporting). **Mark Schwartz:** Conceptualization (equal); Formal analysis (supporting); Writing‐review & editing (supporting). **John Williams:** Conceptualization (equal); Formal analysis (supporting); Methodology (equal); Supervision (lead); Writing‐review & editing (lead).

## Supporting information

Supplementary Material

## Data Availability

All data used in the analyses of this study are included in the Supplementary Material or in the online data included in the main or supporting information for Williams et al. (2018; https://onlinelibrary.wiley.com/doi/full/10.1111/jbi.13413) that was the source of the climate exposure data used in this study.
