# Supplementary material for: Trait‐based climate vulnerability of native rodents in southwestern Mexico
Source: Ecol Evol. 2020 May 18;10(12):5864–76. doi: 10.1002/ece3.6323 (PMC7319118; doi:10.1002/ece3.6323)
Supplement: Supplementary file 1 — Supplementary Material [file ECE3-10-5864-s001.docx]

**Title:** Trait-based climate vulnerability of native rodents in southwestern Mexico

**SUPPORTING INFORMATION**

Appendix S1. Values for each metric of sensitivity and adaptive capacity to climate change for rodent species evaluated in this study. Habitat unsuitability represents the percentage of a species´ distribution range in Oaxaca State (Mexico) located in unfavorable habitats (permanent agricultural fields, urban zones, and bare lands). The distribution range is expressed as percentage of national territory. * = Endemic to Oaxaca

|  | SENSITIVITY | | | ADAPTIVE CAPACITY | |
| --- | --- | --- | --- | --- | --- |
| Species | Habitat suitability  (%) | Elevation range  (m) | Distribution range in Mexico (%) | Generation (d) length  (days) | Mean Weight (g) WWeight  (gr) |
| Baiomys musculus | 6.5 | 2000 | 15.9 | 405 | 9 |
| Coendou mexicanus | 10.5 | 2350 | 20.5 | 3022 | 2000 |
| Cuniculus paca | 23.4 | 1800 | 14.1 | 2098 | 8172 |
| Dasyprocta mexicana | 26.8 | 600 | 2.8 | 1891 | 4999 |
| Dipodomys phillipsii | 3.2 | 1950 | 9.6 | 825 | 41 |
| Glaucomys volans | 3.3 | 2200 | 6.3 | 1930 | 72 |
| Habromys chinanteco* | 0 | 570 | 0.01 | 589 | 40 |
| Habromys ixtlani* | 0 | 500 | 0.01 | 589 | 40 |
| Habromys lepturus* | 0 | 500 | 0.01 | 589 | 85 |
| Heteromys desmarestianus | 17.8 | 1815 | 10.1 | 874 | 74 |
| Liomys irroratus | 2.7 | 3050 | 27.8 | 874 | 49 |
| Liomys pictus | 10.1 | 2045 | 20.8 | 874 | 43 |
| Megadontomys cryophilus* | 2.8 | 1100 | 0.3 | 589 | 57 |
| Megadontomys thomasi | 1.7 | 500 | 2.5 | 766 | 110 |
| Microtus mexicanus | 4.3 | 1895 | 26.9 | 374 | 35 |
| Microtus oaxacensis* | 0 | 1000 | 0.03 | 374 | 36 |
| Microtus quasiater | 0.8 | 1450 | 1.4 | 374 | 40 |
| Microtus umbrosus* | 0.4 | 700 | 0.04 | 374 | 42 |
| Neotoma mexicana | 8 | 4045 | 56.4 | 857 | 203 |
| Nyctomys sumichrasti | 14 | 1500 | 12.1 | 589 | 60 |
| Oligoryzomys fulvescens | 9.7 | 1550 | 30.1 | 589 | 25 |
| Orthogeomys Cuniculus* | 26.7 | 30 | 0.01 | 872 | 500 |
| Orthogeomys grandis | 8.9 | 1700 | 4.8 | 872 | 500 |
| Orthogeomys hispidus | 22.7 | 2360 | 13.2 | 872 | 500 |
| Oryzomys alfaroi | 11 | 1490 | 11.4 | 319 | 33 |
| Oryzomys chapmani | 5.5 | 950 | 2 | 319 | 50 |
| Oryzomys couesi | 9.9 | 2300 | 45.1 | 319 | 69 |
| Oryzomys melanotis | 10 | 2000 | 6.5 | 319 | 50 |
| Oryzomys rostratus | 13.1 | 1500 | 9.8 | 319 | 42.5 |
| Peromyscus aztecus | 7.4 | 2700 | 7.5 | 635 | 34 |
| Peromyscus difficilis | 3.1 | 2500 | 25.1 | 635 | 28 |
| Peromyscus furvus | 2.8 | 2300 | 1.2 | 635 | 33 |
| Peromyscus gratus | 3.8 | 990 | 23.8 | 635 | 27 |
| Peromyscus leucopus | 13 | 3000 | 33.3 | 635 | 18 |
| Peromyscus maniculatus | 2.3 | 3740 | 47.8 | 940 | 20 |
| Peromyscus megalops | 6.2 | 1500 | 1.3 | 635 | 66 |
| Peromyscus melanocarpus* | 0.9 | 1900 | 0.4 | 635 | 59 |
| Peromyscus melanophrys | 5.8 | 2650 | 26.1 | 635 | 40 |
| Peromyscus melanurus* | 1.8 | 1200 | 0.2 | 635 | 40 |
| Peromyscus mexicanus | 12.6 | 1400 | 11.8 | 635 | 43 |
| Reithrodontomys fulvescens | 6 | 2600 | 64.1 | 203 | 12 |
| Reithrodontomys megalotis | 7.5 | 4000 | 43.5 | 203 | 11 |
| Reithrodontomys mexicanus | 7.1 | 1710 | 10.8 | 203 | 16 |
| Reithrodontomys microdon | 2.4 | 825 | 3 | 203 | 20 |
| Reithrodontomys sumichrasti | 5.3 | 2400 | 13.2 | 203 | 19 |
| Rheomys mexicanus* | 14.3 | 2200 | 0.2 | 589 | 40 |
| Sciurus aureogaster | 9.9 | 3300 | 30 | 2122 | 456 |
| Sciurus deppei | 15.2 | 2800 | 18.8 | 2122 | 250 |
| Scotinomys teguina | 0.3 | 1940 | 1.5 | 589 | 12 |
| Sigmodon alleni | 8.9 | 3050 | 8.3 | 597 | 174 |
| Sigmodon hispidus | 3 | 3050 | 67.8 | 597 | 111 |
| Sigmodon leucotis | 3.7 | 823 | 13.6 | 597 | 132 |
| Sigmodon mascotensis | 7.5 | 2550 | 12.2 | 597 | 120 |
| Spermophilus variegatus | 2.5 | 3600 | 60.6 | 1054 | 715 |
| Tylomys nudicaudus | 9.9 | 1600 | 11.3 | 695 | 182 |

Appendix S2. Percentage of species´ distribution range located in cells projected to be exposed (in the “high”, “very high” and “non-analog” categories in Williams et al.´s (2018) study) under the lower impact (CNRM-RCP 4.5) and higher impact (MPI-RCP 8.5) climate scenarios, for two future periods: near-future (2015-2039) and end-century (2075-2099). *=Endemic to Oaxaca

|  | CNRM RCP 4.5 | | MPI RCP 8.5 | |
| --- | --- | --- | --- | --- |
| Species | 2015-2039 | 2075-2099 | 2015-2039 | 2075-2099 |
| Baiomys musculus | 14.1 | 10.4 | 16.2 | 62.3 |
| Coendou mexicanus | 8.3 | 13.6 | 21.8 | 64.8 |
| Cuniculus paca | 12.8 | 20 | 37.9 | 83.8 |
| Dasyprocta mexicana | 11.3 | 19.5 | 44.4 | 90.4 |
| Dipodomys phillipsii | 30.1 | 16.9 | 14.1 | 67.8 |
| Glaucomys volans | 10.7 | 3.6 | 7.2 | 43.2 |
| Habromys chinanteco* | 16.8 | 31.6 | 22.1 | 16.8 |
| Habromys ixtlani* | 38.5 | 50 | 35.6 | 10.6 |
| Habromys lepturus* | 7.3 | 7.3 | 6.3 | 17.7 |
| Heteromys desmarestianus | 13.8 | 24.6 | 39.4 | 81.7 |
| Liomys irroratus | 17 | 6.4 | 9.4 | 48.2 |
| Liomys pictus | 7.8 | 12.8 | 20 | 70.7 |
| Megadontomys cryophilus* | 11.2 | 14.5 | 10.9 | 35.1 |
| Megadontomys thomasi | 22.6 | 3 | 11.8 | 69.9 |
| Microtus mexicanus | 12.2 | 12.6 | 15 | 46.4 |
| Microtus oaxacensis* | 13.4 | 20.6 | 14.1 | 24.6 |
| Microtus quasiater | 18.2 | 32.3 | 27.1 | 54.1 |
| Microtus umbrosus* | 7.2 | 11.2 | 9.6 | 28.4 |
| Neotoma mexicana | 14.2 | 12.7 | 19.4 | 64.6 |
| Nyctomys sumichrasti | 7.8 | 13.8 | 24.6 | 73 |
| Oligoryzomys fulvescens | 12.7 | 10.3 | 20.1 | 65.4 |
| Orthogeomys cuniculus* | 9.3 | 29.5 | 54.5 | 100 |
| Orthogeomys grandis | 13.2 | 8.1 | 17.6 | 70.6 |
| Orthogeomys hispidus | 13.7 | 22.4 | 42.5 | 83.5 |
| Oryzomys alfaroi | 7 | 9.5 | 18.8 | 60.8 |
| Oryzomys chapmani | 15.6 | 27.5 | 30.2 | 61.6 |
| Oryzomys couesi | 13.8 | 12.4 | 21.7 | 66.6 |
| Oryzomys melanotis | 3.4 | 6.3 | 13.5 | 72.5 |
| Oryzomys rostratus | 11.2 | 17.7 | 28 | 66.4 |
| Peromyscus aztecus | 14.1 | 12 | 18.7 | 58.4 |
| Peromyscus difficilis | 20.7 | 9 | 7.9 | 44.5 |
| Peromyscus furvus | 15.6 | 26.4 | 20 | 37.2 |
| Peromyscus gratus | 25.8 | 9.1 | 8.2 | 49.3 |
| Peromyscus leucopus | 11 | 17.4 | 26.1 | 63.8 |
| Peromyscus maniculatus | 16.9 | 18.9 | 14.8 | 46.3 |
| Peromyscus megalops | 11.4 | 3.7 | 7.8 | 43.5 |
| Peromyscus melanocarpus* | 16.1 | 24.1 | 21.6 | 45.8 |
| Peromyscus melanophrys | 17.5 | 13.9 | 19 | 63.9 |
| Peromyscus melanurus* | 3.6 | 4.9 | 5.7 | 56.8 |
| Peromyscus mexicanus | 7.5 | 13.2 | 23.7 | 70.8 |
| Reithrodontomys fulvescens | 15.1 | 11.3 | 17 | 61.6 |
| Reithrodontomys megalotis | 18.1 | 13.2 | 21.9 | 61.7 |
| Reithrodontomys mexicanus | 10.2 | 15.4 | 20.7 | 57 |
| Reithrodontomys microdon | 22.2 | 11.7 | 10.9 | 40.6 |
| Reithrodontomys sumichrasti | 11.1 | 10 | 13.9 | 56.7 |
| Rheomys mexicanus* | 8.6 | 19.1 | 26.9 | 62.7 |
| Sciurus aureogaster | 13.8 | 12.4 | 21.7 | 66.6 |
| Sciurus deppei | 13.8 | 23.6 | 36.9 | 77 |
| Scotinomys teguina | 8.7 | 14.4 | 14.3 | 57.4 |
| Sigmodon alleni | 3.5 | 4.9 | 11.2 | 65 |
| Sigmodon hispidus | 16.1 | 13.8 | 22.7 | 63.6 |
| Sigmodon leucotis | 28.9 | 7.3 | 14.4 | 60.5 |
| Sigmodon mascotensis | 11.6 | 9 | 16.9 | 64.3 |
| Spermophilus variegatus | 38.7 | 21.5 | 18.1 | 79.6 |
| Tylomys nudicaudus | 11.3 | 13.1 | 20.6 | 63.8 |

Appendix S3. Climate change vulnerability scoring for the lower impact (CNRM-RCP 4.5 scenario) and higher impact (MPI-RCP 8.5) climate scenarios for near-future (2015-2039) and end-century (2075-2099) time periods. HV = highly vulnerable; PP = potential persisters; PA = potential adapters; HLR = high latent risk; LV = low vulnerability; EO = exposed only; SO = sensitive only; LACO = low adaptive capacity only. * = Endemic to Oaxaca.

|  | CNRM-RCP 4.5 | | MPI-RCP 8.5 | |
| --- | --- | --- | --- | --- |
| Species | 2015-2039 | 2075-2099 | 2015-2039 | 2075-2099 |
| Baiomys musculus | LACO | LACO | LACO | PP |
| Coendou mexicanus | LACO | LACO | LACO | PP |
| Cuniculus paca | LACO | LACO | LACO | PP |
| Dasyprocta mexicana | HLR | HLR | HLR | HV |
| Dipodomys phillipsii | LACO | LACO | LACO | PP |
| Glaucomys volans | LACO | LACO | LACO | LACO |
| Habromys chinanteco* | HLR | HLR | HLR | HLR |
| Habromys ixtlani* | HLR | HLR | HLR | HLR |
| Habromys lepturus* | SO | SO | SO | SO |
| Heteromys desmarestianus | LACO | LACO | LACO | PP |
| Liomys irroratus | LACO | LACO | LACO | LACO |
| Liomys pictus | LACO | LACO | LACO | PP |
| Megadontomys cryophilus* | SO | SO | SO | SO |
| Megadontomys thomasi | SO | SO | SO | PA |
| Microtus mexicanus | LACO | LACO | LACO | LACO |
| Microtus oaxacensis* | HLR | HLR | HLR | HLR |
| Microtus quasiater | HLR | HLR | HLR | HLR |
| Microtus umbrosus* | SO | SO | SO | SO |
| Neotoma mexicana | LACO | LACO | LACO | PP |
| Nyctomys sumichrasti | LV | LV | LV | EO |
| Oligoryzomys fulvescens | LACO | LACO | LACO | PP |
| Orthogeomys cuniculus* | HLR | HLR | HLR | HV |
| Orthogeomys grandis | HLR | HLR | HLR | HV |
| Orthogeomys hispidus | LACO | LV | LV | PP |
| Oryzomys alfaroi | LACO | LACO | LACO | PP |
| Oryzomys chapmani | SO | SO | SO | PA |
| Oryzomys couesi | LV | LV | LV | EO |
| Oryzomys melanotis | LV | LV | LV | EO |
| Oryzomys rostratus | LV | LV | LV | EO |
| Peromyscus aztecus | LACO | LACO | LACO | LACO |
| Peromyscus difficilis | LACO | LACO | LACO | LACO |
| Peromyscus furvus | HLR | HLR | HLR | HLR |
| Peromyscus gratus | HLR | HLR | HLR | HLR |
| Peromyscus leucopus | LACO | LACO | LACO | PP |
| Peromyscus maniculatus | LACO | LACO | LACO | LACO |
| Peromyscus megalops | SO | SO | SO | SO |
| Peromyscus melanocarpus* | SO | SO | SO | SO |
| Peromyscus melanophrys | LACO | LACO | LACO | PP |
| Peromyscus melanurus* | HLR | HLR | HLR | HLR |
| Peromyscus mexicanus | LV | LV | LV | EO |
| Reithrodontomys fulvescens | LACO | LACO | LACO | PP |
| Reithrodontomys megalotis | LACO | LACO | LACO | PP |
| Reithrodontomys mexicanus | LACO | LACO | LACO | LACO |
| Reithrodontomys microdon | HLR | HLR | HLR | HLR |
| Reithrodontomys sumichrasti | LACO | LACO | LACO | LACO |
| Rheomys mexicanus* | HLR | HLR | HLR | HV |
| Sciurus aureogaster | LACO | LACO | LACO | PP |
| Sciurus deppei | LACO | LACO | LACO | PP |
| Scotinomys teguina | HLR | HLR | HLR | HLR |
| Sigmodon alleni | LV | LV | LV | EO |
| Sigmodon hispidus | LV | LV | LV | EO |
| Sigmodon leucotis | SO | SO | SO | PA |
| Sigmodon mascotensis | LV | SO | SO | EO |
| Spermophilus variegatus | LACO | LACO | LACO | PP |
| Tylomys nudicaudus | LV | LV | LV | EO |

Appendix S4. Sensitivity analysis of vulnerability thresholds (see Methods for description of criteria). The table below examines the effect of varying the threshold value used for each of the criteria used to assess the climate vulnerability of the 55 rodent species of Oaxaca, Mexico that are evaluated in this study. The number in the parenthesis represents the percentage of species in each category. Under the lenient scenario (column 2) thresholds are relaxed to make it easier for a species to be classified as vulnerable, while under the stricter scenario (column 4), thresholds are more rigorous and require more severe conditions for vulnerability to be established. Thresholds used in this study (“Existing”) are presented in the column 3. Climate exposure values are based on the end-century higher impact (MPI-RCP 8.5) scenario.

| Vulnerability criteria | Lenient scenario | Existing | Stricter scenario |
| --- | --- | --- | --- |
| Climate exposure | ≥45 % (80 %) | ≥60 % (60 %) | ≥75 % (12.7 %) |
| (% Oax cells in category) |  |  |  |
| Habitat unsuitability | ≥45 % (0) | ≥60 % (0) | ≥75 % (0) |
| (% cells in Oaxaca) |  |  |  |
| Distribution range | ≤10 % (47.3 %) | ≤5 % (34.5 %) | ≤1 % (18.2 %) |
| (% of Mexican territory) |  |  |  |
| Elevation range (m) | ≤1500 (36.4) | ≤1000 (21.2 %) | ≤500 (7 %) |
| Weight (g) | ≤80 (69.1%) | ≤40 (38.2 %) | ≤20 (16.4%) |
| Generation length (days) | ≥500 (72.7 %) | ≥800 (23.6 %) | ≥1100 (12.7 %) |

Appendix S5. Number of species (relative proportion is presented in parenthesis) under the different categories of vulnerability obtained in the threshold sensitivity analysis (see Appendix S4). Results from the thresholds used in this study are in column 3 (“Existing”).

| Vulnerability category | Lenient thresholds | Existing | Stricter thresholds |
| --- | --- | --- | --- |
| Highly vulnerable | 20 (36.4 %) | 4(7.3 %) | 0 |
| High latent risk | 11 (20 %) | 9 (16.4 %) | 0 |
| Potential persisters | 23 (42 %) | 17 (31 %) | 4 (7.3 %) |
| Potential adapters | 0 | 3 (5.5 %) | 1(1.8 %) |
| Low adaptive capacity only | 1(1.8 %) | 8 (14.5 %) | 12 (21.8 %) |
| Exposed only | 0 | 10 (18.2 %) | 2 (3.2 %) |
| Sensitive only | 0 | 4 (7.3 %) | 10 (18.2 %) |
| Low vulnerability | 0 | 0 | 26 (47.3 %) |
